# Positive Participatory Organizational Interventions: A Multilevel Approach for Creating Healthy Workplaces

**DOI:** 10.3389/fpsyg.2021.696245

**Published:** 2021-06-28

**Authors:** Karina Nielsen, Marit Christensen

**Affiliations:** ^1^Institute of Work Psychology, The University of Sheffield, Sheffield, United Kingdom; ^2^Norwegian University of Science and Technology, Trondheim, Norway

**Keywords:** participatory interventions, IGLO model, resources, JD-R, healthy workplaces, positive psychology

## Abstract

In the following perspective paper, we argue for the importance of conducting research on positive participatory organizational interventions. We propose that these types of interventions are important because they not only focus on eliminating or reducing adverse job demands but focus also on developing job resources. To achieve the best effects, actions should be taken to address demands and resources at the individual, group, leader and organizational levels. We furthermore suggest that the participatory intervention process itself may also build resources at these four levels.

## Introduction

According to the World Health Organization (WHO), healthy workplaces are characterized by employees and managers collaborating through a continuous improvement process to protect and promote the health, safety, and well-being of employees and the sustainability of the workplace (World Health Organization and Burton, 2010). Furthermore, the WHO defines health as “a state of complete physical, mental, and social well-being and not merely the absence of disease or infirmity” (WHO Definition of Health, 2015). To create healthy workplaces, it is therefore crucial to also develop the positive aspects of the job and build workers' personal resources to ensure good health among employees (Christensen et al., 2017). Despite a few promising examples (Christensen et al., 2019), there are, however, few intervention studies that focus explicitly of how to build healthy workplaces taking a positive psychology approach. Our perspective paper aims to apply lessons learned from the wider intervention literature regarding participatory multilevel approaches that could be applied to the field of positive participatory interventions (PPOIs).

PPOIs focus on improving the work environment and employee well-being. Such improvements happen through changing work policies, practices, and procedures through a collaborative approach where managers and employees jointly decide on the process (the design and implementation of the intervention) and the content of the intervention (changes to work policies, practices, and procedures) (Nielsen and Noblet, 2018). PPOIs employ a problem solving cycle approach to change and go through the phases of (1) preparation, (2) screening, (3) action planning, (4) implementation of action plans and (5) evaluation (Nielsen and Noblet, 2018).

In this perspective paper, we call for positive psychology researchers to create healthy workplaces through the implementation of PPOIs. We suggest the IGLO (Individual, Group, Leader and Organizational levels) framework may be useful to develop the content (action plans and their implementation) and the process (the collaborative process of employees and managers) of such interventions.

## Creating Healthy Workplaces by Changing Work Policies, Practices, and Procedures

In order for PPOIs to achieve their intended outcomes, actions need to be sustainably implemented (von Thiele Schwarz et al., 2020). We argue that the Job Demands Resources model (JD-R) (Demerouti et al., 2001) is a suitable framework for developing actions. The JD-R model has a holistic approach to employee well-being including a motivational process and a health impairment process, respectively activated by resources and demands (Demerouti et al., 2001). Key to the JD-R model is the balance between job demands, i.e., the negative aspects of the job that require sustained effort or skills and job resources, i.e., the positive aspects of the job that enable employees to achieve their goals and stimulate personal growth (Demerouti et al., 2001). When making changes to work policies, practices and procedures it is important to consider this dual perspective and develop and implement action plans that reduce or eliminate job demands and build resources to not only reduce poor health but also to enable employees to grow and thrive in their jobs. Building resources is particularly important as resources may help buffer those aspects of the job that are not easily eliminated or reduced (Vignoli et al., 2017).

PPOIs focus on changing work practices, policies and procedures, and prescribe a structured, organizational process (Nielsen, 2013). Action plans should address both job demands and resources and thus focus on both the positive and negative aspects at work (Christensen et al., 2017). We propose that demands and resources may be addressed at multiple levels in the organization (Nielsen and Noblet, 2018). Recently, Nielsen and Miraglia (2017) and Day and Nielsen (2017) suggested the IGLO framework for building resources to promote positive employee and organizational health at the Individual, Group, Leader and Organizational levels. The IGLO framework can also be applied to job demands. When developing actions, the IGLO framework can be applied at the four levels. We provide brief examples of interventions at these levels, but in reality the possibilities of actions are much wider than presented here.

At the Individual level, we need to address the inherent demands people put on themselves, for example, employees' expectations of how much work they can take on and build individual resources such as beliefs about successfully overcoming challenges at work, i.e., self-efficacy (Bandura, 1986). At the Group level, we need to consider actions that address job demands within the work group, for example, whether group conflict and incivility prevent effective collaboration (Leiter et al., 2012) or whether we need to increase group resources such as effective communication. At the Leader level, leaders at the line management level may engage in abusive leadership or may lack the skills to support employees' development and thus training leaders may be an important way of building leaders' resources in an attempt to reduce adverse demands they place on employees (Kelloway and Barling, 2010).

Finally, at the Organizational level, Human Resource policies and practices may need to be adjusted or developed. Examples of interventions at this level may be jobs designed around high performance work practices, which have been found to be related to employee well-being (Ogbonnaya et al., 2017). High performance practices cover a range of practices across three dimensions. Ability concerns the development of training systems that ensure employees have the necessary skills and competencies to do the job. Motivation concerns the development of clear and transparent career progression pathways and reward systems to motivate workers. Finally, Opportunities refer to designing jobs in a way that encourages teamworking, information sharing, job autonomy, and flexible working (Ogbonnaya et al., 2017).

## Creating Healthy Workplaces Through PPOI Processes

More recently, it has been argued that the process, i.e., how PPOIs are implemented is as important as the content of PPOIs (Nielsen and Miraglia, 2017). The participatory process is key to building organizational resilience, i.e., developing the organization's ability to address adverse job demands and build resources (von Thiele Schwarz et al., 2017).

Multiple benefits of a PPOI process have been identified. First, the process creates employees' buy-in and ownership. Second, it makes use of workers' expertise of which demands and resources need to change and it enables workers to make sense of the PPOI. Third, it optimizes the fit with the organizational context, and it facilitates the PPOI process (Nielsen and Randall, 2012). Fourth, it enables dialogue between managers and workers about what changes need to be made (Christensen et al., 2019). Finally, the dual perspective on both negative and positive aspects of the work environment encourages a balanced understanding of the environment (Christensen et al., 2019).

Although there is a growing awareness of how the intervention process may influence intervention outcomes (Nielsen and Abildgaard, 2013; Nielsen and Randall, 2013), there has to date been limited research on how the participatory process may build resources at the four IGLO levels.

At the individual level, the individual resources relating to Psychological Capital (PsyCap; Brunetto et al., 2020) may be developed. PsyCap consists of four dimensions/resources: Self-efficacy, optimism, hope and resilience. As employees engage in the participatory process, they may build their confidence that they can take on and succeed in addressing adverse working conditions (self-efficacy). The participatory process may also build to employees positive attribution about how their working life may improve as actions to eliminate or reduce demands and build resources are implemented thus increasing their optimism. Equally, the participatory process may build employees' perceptions of hope as they persevere toward achieving the goals of the intervention. Finally, the participatory process may build employees' individual resilience as the review the progress of the implementation of action plans and develop strategies for successfully implementing changes to eliminate or reduce work demands and build resources.

At the group level, the participatory process may build group resources as workers engage in collective decision making about which changes to work practices and procedures to implement. One group-level resource that may be developed through the participatory process is collective sensemaking (Nielsen et al., 2021b). The development of shared understanding occurs through collective sensemaking processes (Maitlis and Christianson, 2014). Employees who engage in the participatory process collectively engage in developing and implementing action plans (Nielsen and Noblet, 2018), meaning they experience similar cues as to how to interpret the situation they find themselves in Goffman (1974), in this case the PPOI process, they are therefore likely to engage in collective sensemaking. The collective exchanges between participants are likely to lead to collectively generated sensemaking as the PPOI progresses (Stensaker et al., 2008). It is possible that through the discussions of working conditions, members of the work team develop a shared meaning of the problems (Weick, 1995). Collective sensemaking around the participatory process has been found to be related to increased work engagement (Nielsen et al., 2021b).

Collective job crafting (Leana et al., 2009) may be another group-level resource which could be developed as a result of the participatory process. As employees collectively explore and agree which changes to work practices, policies, and procedures need to developed and implemented, they craft not only their own jobs, but engage in a collective job crafting process where changes are implemented not only to the benefit of the individual but to the benefit of the entire work group. Hasson et al. (2012) found that in some work groups employees reported more changes had incurred as a result of the PPOI than their line managers reported, despite the managers being officially responsible for implementing changes. This finding could be seen as an indication that employees developed and implemented their own actions.

At the leader level, leaders may develop their own resources as a result of their role in the PPOI. In recent years, there has been an increasing focus on leadership behaviors that promote employee well-being, also termed health promoting leadership (Nielsen and Taris, 2019). Key characteristics of health-promoting leadership is that the leader enact behaviors that support employee well-being. Such behaviors include putting well-being on the agenda in meetings and engaging in discussions with employees about what changes need to be made to ensure their well-being (Franke et al., 2014). The participatory process offers an arena for leaders to put well-being on the agenda and engage in such discussions and thus the process may help leaders develop their health-promoting leadership skills.

At the organizational level, organizational resources may be built in relations to future health and safety management practices. Nielsen et al. (2021a) argued for the integration of PPOI processes into existing practices to ensure sustainable change. In many modern organizations, continuous improvement practices are employed to enhance organizational performance and productivity, the practices focus on continuous improvement of work practices and procedures to streamline the work process (von Thiele Schwarz et al., 2017). One widely used continuous improvement system is that of Lean (Radnor et al., 2012). A key component of Lean is Kaizen, the use of continuous improvement boards that workers employ to identify, implement, evaluate, and integrate the changes to work practices and procedures needed to streamline work processes (Jacobson et al., 2009). von Thiele Schwarz et al. (2017) demonstrated how well-being could be integrated into this process and that as a result the organization built its resources to manage the job demands and resources in a win-win situation.

## Discussion

In this perspective paper, we argue for researchers in positively psychology to create healthy workplaces through multi-level positive participatory organizational interventions PPOIs offer a way forward in creating healthy workplaces in which employees may thrive and develop in their jobs. We argue for a dual perspective approach where PPOIs focus at both eliminating or reducing adverse job demands as well as increasing job resources and that demands and resources need to be addressed at the individual, group, leader and organizational levels (Nielsen and Miraglia, 2017). Based on the IGLO framework, we make two significant contributions. First, the participatory process in itself may build resources at the IGLO levels. We call for research on how the participatory process itself may foster a healthy workplace that empowers employees and facilitates their thriving at work. Second, we argue that action to improve working conditions and well-being should include actions at all IGLO levels.

## Data Availability Statement

The original contributions presented in the study are included in the article/supplementary material, further inquiries can be directed to the corresponding authors.

## Author Contributions

KN developed the first draft. MC contributed with additional text. All authors contributed to the article and approved the submitted version.

## Conflict of Interest

The authors declare that the research was conducted in the absence of any commercial or financial relationships that could be construed as a potential conflict of interest.
